# Evaluation of Spatially Targeted Strategies to Control Non-Domiciliated *Triatoma dimidiata* Vector of Chagas Disease

**DOI:** 10.1371/journal.pntd.0001045

**Published:** 2011-05-17

**Authors:** Corentin Barbu, Eric Dumonteil, Sébastien Gourbière

**Affiliations:** 1 UMR 5244 CNRS/UPVD/EPHE, ‘Biologie et Ecologie Tropicale et Méditerranéenne’, Université de Perpignan Via Domitia, Perpignan, France; 2 Laboratorio de Parasitología, Centro de Investigaciones Regionales “Dr. Hideyo Noguchi”, Universidad Autónoma de Yucatán, Mérida, Yucatan, Mexico; 3 Department of Tropical Medicine, School of Public Health and Tropical Medicine, Tulane University, New Orleans, Louisiana, United States of America; 4 Centre for the Study of Evolution, School of Life Sciences, University of Sussex, Brighton, United Kingdom; Universidad de Buenos Aires, Argentina

## Abstract

**Background:**

Chagas disease is a major neglected tropical disease with deep socio-economical effects throughout Central and South America. Vector control programs have consistently reduced domestic populations of triatomine vectors, but non-domiciliated vectors still have to be controlled efficiently. Designing control strategies targeting these vectors is challenging, as it requires a quantitative description of the spatio-temporal dynamics of village infestation, which can only be gained from combinations of extensive field studies and spatial population dynamic modelling.

**Methodology/Principal Findings:**

A spatially explicit population dynamic model was combined with a two-year field study of *T. dimidiata* infestation dynamics in the village of Teya, Mexico. The parameterized model fitted and predicted accurately both intra-annual variation and the spatial gradient in vector abundance. Five different control strategies were then applied in concentric rings to mimic spatial design targeting the periphery of the village, where vectors were most abundant. Indoor insecticide spraying and insect screens reduced vector abundance by up to 80% (when applied to the whole village), and half of this effect was obtained when control was applied only to the 33% of households closest to the village periphery. Peri-domicile cleaning was able to eliminate up to 60% of the vectors, but at the periphery of the village it has a low effect, as it is ineffective against sylvatic insects. The use of lethal traps and the management of house attractiveness provided similar levels of control. However this required either house attractiveness to be null, or ≥5 lethal traps, at least as attractive as houses, to be installed in each household.

**Conclusion/Significance:**

Insecticide and insect screens used in houses at the periphery of the village can contribute to reduce house infestation in more central untreated zones. However, this beneficial effect remains insufficient to allow for a unique spatially targeted strategy to offer protection to all households. Most efficiently, control should combine the use of insect screens in outer zones to reduce infestation by both sylvatic and peri-domiciliated vectors, and cleaning of peri-domicile in the centre of the village where sylvatic vectors are absent. The design of such spatially mixed strategies of control offers a promising avenue to reduce the economic cost associated with the control of non-domiciliated vectors.

## Author Summary

Chagas disease is one of the most important parasitic diseases in Latin America. Since the 1980's, many national and international initiatives have contributed to eliminate vectors developing inside human domiciles. Today's challenge is to control vectors that are non-adapted to the human domicile, but still able to transmit the parasite through regular short stay in the houses. Here, we assess the potential of different control strategies applied in specific spatial patterns using a mathematical model that reproduces the dynamic of dispersion of such ‘non-domiciliated’ vectors within a village of the Yucatan Peninsula, Mexico. We show that no single strategy applied in the periphery of the village, where the insects are more abundant, provides satisfying protection to the whole village. However, combining the use of insect screens in houses at the periphery of the village (to simultaneously fight insects dispersing from the garden and the forest), and the cleaning of the peri-domicile areas of the centre of the village (where sylvatic insects are absent), would provide a cost-effective control. This type of spatially mixed strategy offers a promising way to reduce the cost associated with the repeated interventions required to control non-domiciliated vectors that permanently attempt to infest houses.

## Introduction

Chagas disease, also called American trypanosomiasis, is caused by the protozoan parasite *Trypanosoma cruzi*, which is primarily transmitted to humans by blood-sucking bugs of the Triatominae subfamily. The disease is endemic throughout Latin America, where it is one of the most important parasitic diseases with large socioeconomic impact. According to various estimates, the prevalence rate in humans varies between 0.1 and 45.2% (with an average of 1.4%), 8 to 15 million people are infected with *T. cruzi* (with 40–50,000 yearly new cases), and 28–75 million individuals are at risk of infection [1]–[3]. The disease causes about 12,500 deaths a year, and is responsible for premature disabilities of workers that are estimated to cost 670,000 disability-adjusted life years lost [4].

Although international initiatives have been launched to reduce transmission of Chagas disease, especially through vector control and screening of blood or organ donors [5], there are still large regions with active vector transmission [6]. One of the main explanations for this is the transmission caused by non-domiciliated triatomines [7]. These vectors are not able to reproduce and develop in the domestic habitat, and thus constitute typical ‘sink’ domestic populations sustained by peri-domestic and/or sylvatic ‘source’ populations [8]. Non-domiciliated vectors tend to jeopardize the efficacy of vector control by insecticide spraying in the domestic habitat because of the re-infestation of treated houses [9], [10], [11]. This situation has been described for several vector species of triatomines as *T. brasiliensis* and *T. pseudomaculata* in Brazil [12], *T. mexicana* in central Mexico [13] and *T. dimidiata* in the Yucatan Peninsula of Mexico and Belize [14], [15]. Accordingly, the risk of transmission associated with non-domiciliated vectors is now identified as a major challenge for the future of Chagas disease control [16], [17], [18], and a key objective is to evaluate the efficacy of classical or alternative control strategies to reduce their abundance.

Identifying optimal strategies can hardly be achieved through laboratory or field experiments, since testing a broad enough number of alternatives would require very large human and financial investments [11], [19]. Alternatively, mathematical models have proven to be very effective at evaluating the relative merit of various alternative strategies to control parasitic diseases [11; and references therein]. In addition, identifying optimal strategies clearly requires a detailed understanding of the vector spatial and temporal infestation dynamics. Valuable insights into such spatio-temporal dynamics can be gained using the framework of meta-population theory combined with presence/absence data [19]–[21]. Although appealing, the use of more elaborated models that include quantitative information on local population sizes requires even more data than the meta-population model *sensus stricto* [22].

In previous contributions, we developed spatially explicit population dynamics models that were able to reproduce and to predict the spatial and temporal dynamics of *T. dimidiata* house infestation observed at the village scale in the Yucatan Peninsula, Mexico. These models provided us with indirect estimates of the origin and characteristics of dispersal of these triatomines [23], [24]. Individuals found inside houses in the Yucatan Peninsula originated in similar proportions from both sylvatic and peri-domestic habitats, dispersed over rather small distances (40–60 m per displacement) and were strongly attracted to houses [24]. Remarkably, the observed and predicted dynamics showed an heterogeneity in transmission risk both in time, with a peak of vector abundance during March–June [14], [25], and in space, with much higher abundance of insects in the periphery of the village reflecting the influence of the sylvatic habitat [11], [26]. The temporal optimization of insecticide spraying with respect to this pattern has already been investigated at the scale of one house [11], but the spatial micro-scale heterogeneity suggests that interventions could also be spatially targeted. Such interventions would focus on the periphery of the village, where bugs were found more abundant. While temporal heterogeneity adds constraints on control strategies (i.e. the timing of intervention has to match the seasonality of house infestation, [11]), spatial heterogeneity could have beneficial consequences for control activities as it might allow to reduce the overall surface (or number of houses) to be treated and thus allow to reduce the cost associated with control. Properly assessing whether such spatial design is relevant requires evaluating not only the efficacy of control in the treated areas, but also the impact of the control interventions in the untreated areas of the same village.

In this contribution, we aimed to build on our understanding of the temporal optimization of control strategies [11], as well as our previous spatial modelling [23], [24] to evaluate the potential of several strategies. We first focus on conventional strategies — namely indoor insecticide spraying, use of door/window insect screens and peri-domicile management — that have been used to control vectors of different diseases as well as *T. dimidiata* [27]–[29]. We further look at the potential of insect lethal traps that are currently extensively investigated for the control of a variety of vector species [30], [31]. Finally, since we have previously found that *T. dimidiata* was directly attracted to houses [24], a control alternative could be to eliminate this house attractiveness, and the potential of such a strategy was also explored.

## Materials and Methods

### General approach

We aimed to set up a spatial population dynamics model able: (1) to reproduce and predict the temporal variations of vector abundance in all the houses of one village in the absence of control, and (2) to spatially represent various control strategies. We adapted previous population dynamic models [23], [24], and combined them with a mathematical description of the control strategies that we aimed at evaluating. The resulting model predicts the temporal variations in vector abundance in every house of the village as a function of survival, reproduction and dispersal of the triatomines, and the effect of the above control strategies on the demographic processes at each point of the village. It was then used for the evaluation of the efficacy of spatially targeted interventions based on each of those strategies. Model predictions in absence of control were fitted through a maximum likelihood approach to a first set of spatio-temporal data describing house infestation dynamics by *T. dimidiata* within a village in the absence of vector control. We tested the predictive value of the resulting parameterized model on a replicate data set, corresponding to the infestation dynamics observed in the same village the following year.

The description of the effect of the different control strategies was then added to the model, and the resulting framework was used to explore the efficacy of control interventions whose spatial coverage was progressively increased from the border to the centre of the village. The efficacy of each intervention was evaluated as the percentage of reduction in the yearly abundance of vectors in the village, in comparison with the expected abundance in the absence of control intervention that we evaluated from the model with no control. Efficacy was also related to the consented effort, as measured by the number of households, where control strategies were applied (either in the domestic or peri-domestic habitats). We performed a sensitivity analysis to each survival, reproduction or dispersal parameter of the model to ensure the robustness of our conclusions on the efficacy of the various interventions within the confidence region associated with the maximum likelihood estimate of model parameters. We further conducted a sensitivity analysis to different parameters of the model that described the efficacy of each of the strategies as measured by their impact on the survival, reproduction or dispersal of triatomines.

### Spatial and temporal abundance data sets

The spatio-temporal pattern of house infestation was observed in the rural village of Teya, Yucatan, Mexico over a two-year period from August 2006 to October 2008 [26]. All houses were identified and geo-referenced with a handheld global positioning system (GPS). Insects were collected by a standardized methodology based on community participation [32], and data were imported into a geographic information system (GIS) database (ArcView 3.2 -Environmental Systems Research Institute, Redlands, CA, USA) to produce maps of observed triatomine abundance in the houses over 2-week intervals [32]. Participating families provided oral consent prior to their participation, as written consent was waived because the study involved no procedures for which written consent is normally required outside of the research context. Consent was logged in field notebooks. All procedures, including the use of oral consent, were approved by the Institutional Bioethics Committee of the Regional Research Centre “Dr. Hideyo Noguchi”, Universidad Autonoma de Yucatan.

### Spatially explicit population dynamic model with no control

We set up a GIS-based Spatially Explicit Model (GIS-SEM) as such modelling provides a suitable framework to investigate spatial population dynamics in real landscapes by importing GIS data on a grid representing the area under study [33]. Our GIS-SEM model was based on Cellular Automaton (CA) formalism [34]. It consisted of a grid of cells representing the village of Teya, and allowed the calculation of the temporal variations of the vector abundance in cells, referred to as *state variables*, according to both *local rules* describing birth and death processes of bugs within cells, and *dispersal rules* that allow accounting for walking and flight movements between neighboring cells. This model was similar in essence to the models built by Barbu et al. [24], but with two necessary adaptations. First, the *local* and *dispersal rules* were described in a deterministic rather than stochastic manner to reduce the complexity of the model and shorten the simulation time. Second, the time unit of the model was changed from 15 days to a day to allow specifying the effect of control on a daily basis.

A deterministic CA such as the one intended here is defined as a quadruple *Q* = (*A*,*S*,*V*,*f*), where *A* is the grid of cells arranged uniformly to represent the studied area; *S* is the set of values that can be taken by the state variables; *V* is the neighborhood function that allows identifying the set of neighboring cells *V*(*c*) that contribute to the change of the state variable of any given cell *c* by the mapping:
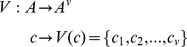
(1)with *v* denoting the size of the neighborhood; and where *f* is the function describing the *local* and *dispersal rules* and thus specifies how the set of neighboring cells *V*(*c*) changes the state of the cell *c* from one time step to another:
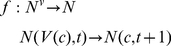
(2)with *N*(*c*,*t*), the *state variable* that tracks the status of cell *c* at time *t*.

#### Definition of the grid of cells A representing the study area

**Figure 1:**
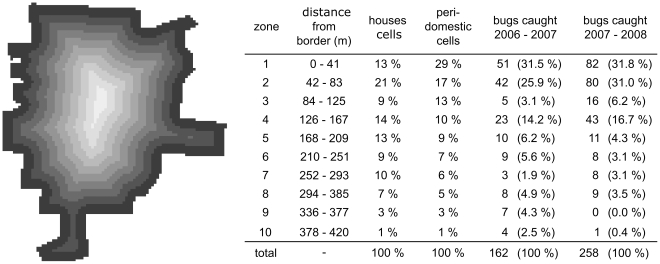
Grid of the village of Teya, Yucatan (Mexico) and the control zones. The grid was derived from the satellite image and the GPS coordinates of all houses. Each cell of the grid corresponds to a surface of 13.5×13.5 m. The ten concentric zones that were used to test the efficacy of spatially targeted control appear on the map. The table gives the proportion of houses, the proportion of peri-domiciles and the number of bugs found (in the absence of control) between mid-September 2006 and 2007, and between mid-September 2007 and 2008, in each of these zones.

A raster map of 88×104 pixels was derived from a satellite image and combined with the GPS coordinates of all the houses to produce a grid that provided a spatial description of the village of Teya as well as a basic description of the forest habitat by a single layer of cells surrounding the village, as previously described [24]. Cells of size 13.5×13.5 m were classified into four different types corresponding to domestic (houses), peri-domestic (yards and streets around the houses), border of the village, and sylvatic habitat (bushes, forest and agricultural land around the village). We referred to these different subsets of cells as *A_d_* (480 cells), *A_p_* (4,847 cells), *A_b_* (466 cells), and *A_s_* (3,359 cells), respectively. The distribution of the domestic and peri-domestic cells within the village is described in Figure 1.

#### Definition of the set of values S for the state variables

To allow a deterministic description of the dynamic, state variables were defined as a continuous version of the discrete state variables used in our previous model [24]. We followed the number of individuals in each set of domestic (*A_d_*) and peri-domestic (*A_p_*) cells, but did not track the number of individuals in the sets of cells representing the border (*A_b_*) or the outside of the village (*A_s_*). Given the lack of abundance data on colonies in peri-domestic area, we focused on the abundance of dispersers, i.e. individuals that have left colonies located in the peri-domestic and sylvatic habitats and are dispersing in the village. As in many similar source-sink models [22], sources (colonies) were assumed to produce dispersing individuals at a constant rate (see below). Finally, we only modelled the adult part of the population since it accounts for over 90% of the triatomines found inside houses [24]. Accordingly, we defined *N*(*c*,*t*) as the density of dispersing adult triatomines in cell *c* at time *t*, with *c*∈*A_d_*∪*A_p_* (the domestic and peri-domestic cells). These state variables took values on *S* = ℜ^+^.

#### Definition of the neighborhood function V

Vectors present in a cell contributed to changes in vector abundance in other cells through dispersal. As before [24], we modelled immigration from the forest surrounding the village by a simple input of bugs located at the forest border. Accordingly, the set of cells a vector can disperse from is *A_d_*∪*A_p_*∪*A_b_*. In addition, vectors have been shown to disperse over rather small average distances [24], so that *V*(*c*), the set of neighboring cells that contributed to change the status of a given cell *c*, was defined to account for a restricted range of dispersal:
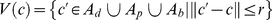
(3)where *r* is the size of the dispersal range.

#### Definition of the function f including the local and dispersal rules

Function *f* accounted for vector demography and dispersal within the village when no control strategies were applied. It was subdivided into *local rules* describing the survival or death of bugs within each cell, and *dispersal rules* that allowed accounting for dispersal from colonies and walking/flight movements between cells. The *local rules* involved survival of adults in the domestic habitats (*S_d_* – probability per time unit) and peri-domestic habitats (*S_p_* – probability per time unit). For simplicity, reproduction in the domestic habitat was neglected since house infestation is associated with a limited or virtually null fertility [8], [11], [25]. Reproduction in the peri-domestic habitat was accounted for, but only by considering those cells as fixed ‘sources’ [35], [36], where individuals are born and can disperse from; consequently, this process was modelled via the *dispersal rules* (see the definition of *K_p_* below) [24]. The number of dispersing adults after the death process *N*(*c*,*t*+*τ*) can then be written for every cell 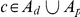:
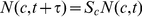
(4)where *S_c_*∈{*S_d_*, *S_p_*}. *τ* is defined to apply the local and dispersal rules in a sequential way, and does not take a specific time value.

*Dispersal rules* were based on previous estimates derived from the comparison of stochastic models, through a selection model approach [37], [38]. To ensure the validity of these estimates with the current deterministic model, a similar selection model approach was performed with analogous deterministic models and provided similar results (data not shown). The most supported *dispersal rules* were defined by two sets of parameters [24]. First, the number of individuals leaving colonies of the peri-domestic (*K_p_* – number of insects per time unit) and sylvatic (*K_s_* – number of insects per time unit) habitats to join the pool of dispersers and a rate of flight/walk initiation (*d* – probability per time unit) for those individuals that already are in the pool of dispersers [24]. Second, a distribution of dispersal distance that combined a zero-truncated Gaussian distribution with a modal distance (*D* – number of meters per displacement) and a standard deviation (*σ* – number of meters per displacement), and a parameter *H* used to weight the zero-truncated Gaussian distribution according to the type of cells considered [24]. *H* was a ratio measuring the relative attractiveness of houses to bugs, as compared to the attractiveness of the peri-domestic and sylvatic habitats for an equal surface. It was set to 1 for both the peri-domestic and sylvatic habitats and was ≥1 for the domestic habitat.

Those two sets of parameters allowed to fully specify the set of cells *V*(*c*) by cutting off dispersal distances that collectively accounted for less than 1% probability, formally defining the value of *r* appearing in equation 3. For each departure cell *c'*, this restricted set of weighted probabilities was then normalized so that dispersal probabilities add up to one when summed over all possible arrival cells *c*. The *dispersal rules* then simply read:
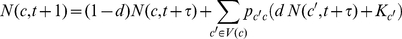
(5a)where *p_c'c_* stands for the normalized probabilities of dispersal from cell *c'* to *c*, and 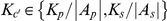, where 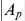 and 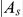 denote the number of cells in sets *A_p_* and *A_s_*. Since individuals were not numbered in the forest habitat, individuals reaching or crossing the border of the village disappeared according to standard absorbing boundary conditions.

Finally, to mimic the seasonal dispersal of *T. dimidiata*, which mostly takes place during a three months' ‘immigration period’ in spring [8], [11], [14], [25], equation 5a was applied only from March 16 to June 15 in Year 1 and from April 1 to July 1 in Year 2 (according to the observations made during these two years). During the rest of the year, vectors were assumed not to leave colonies to join the dispersing pool, although already dispersing individuals were still allowed to move between cells. During this non-immigration period, *K_p_* = *K_s_* = 0, and the equation 5a was replaced by:
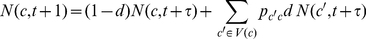
(5b)


The function *f* in the absence of control was then obtained by applying sequentially equations 4 and 5 (a or b), and was used to predict the spatial and temporal population dynamics within the village by calculating the number of bugs at time *t*+1 (e.g., *N*(*c*,*t*+1)) as a function of those numbers at time *t* (e.g., *N*(*c*,*t*)), for any 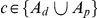. Changes in cells' status, from *N*(*c*,*t*) to *N*(*c*,*t*+*τ*) or from *N*(*c*,*t*+*τ*) to *N*(*c*,*t*+1) were evaluated simultaneously from the earliest states of all cells.

### Parameter estimates of the model with no control

Maximum likelihood estimates (MLE) of the parameters of the model with no control were obtained using the spatio-temporal data sets describing *T. dimidiata* infestation dynamics of the village of Teya between mid-September 2006 and mid-September 2007. Model predictions were fitted to the observed number of bugs in each cell of the 24 maps describing the average biweekly distribution within the village. The log likelihood (LLH) value was then calculated as follows:
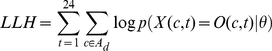
(6)where log denotes the natural logarithm, *X*(*c*,*t*) is the statistical variable corresponding to the number of adults in cell *c*, *O*(*c*,*t*) the observed abundance in this cell, and *θ* is a set of parameters of the model. Probabilities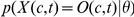 were defined assuming a zero-inflated Poisson distribution to take into account an excess of null abundance in the data set [39], possibly due to the non-participation of a proportion (*w*) of householders, with *w* = 0.7 as before [24].

The parameters *θ* of the model were identified using a genetic algorithm run at the super-computing centre ‘Institut du Développement et des Ressources en Informatique Scientifique (IDRIS)’ located at Orsay, France (http://www.idris.fr/ - Project IDRIS 112290). Genetic algorithms search for solutions using techniques inspired by natural evolution. The interested reader can find a detailed description of such methods and the typical terminology we adopted below in [40]. The algorithm considered the 8 parameters of the model (*S_d_*, *S_p_*, *d*, *K_p_*, *K_s_*, *D*, *σ* and *H*) to be estimated as independent quantitative traits with a continuum of alleles representing possible trait values within biologically relevant domains. The fitness function corresponded to the LLH value defined with respect to the GIS-SEM model with no control described above. The fittest individuals were selected to produce offspring through free recombination and unbiased mutations. The variance of the effect of the mutations was dynamically adapted to the variance in the parental population. All codes were written in C/MPI.

Confidence intervals were calculated by establishing the profile likelihood for each parameter 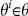, and by using these relationships to determine the 1−*α* confidence region 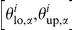 defined as:
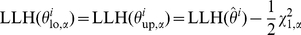
(7)where 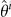 is the MLE of parameter 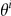 and 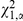 stands for the (1−*α*)^th^ quantile of the 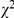 distribution on 1 degree of freedom [41].

### Predictive quality of the model with no control

The ability of the parameterized model to predict other infestation dynamics was tested by comparing its prediction to the spatio-temporal distribution of bug abundance in a second year of infestation of the same village. A Poisson regression between observed and predicted abundances was performed after data were pooled over 3-month periods (starting in mid-September) and within three distance categories: 0–80 m, 81–200 m and >200 m from the bush area outside the villages [24], [26]. The McFadden's likelihood ratio index was used as a pseudo R-squared.

### Evaluation of spatially targeted control strategies

Because the spatial distribution of bugs follows a spatial gradient with higher abundance at the periphery of the village [24], [26], the control strategies were applied to a ring of cells located at the border of the village, the size of this ring increasing progressively until the intervention covered the whole village (Figure 1). The efficacy of any given spatially targeted strategies was measured in terms of yearly bug abundance both in the whole village and in the different concentric rings. This allowed us to quantify the relationship between the effort in terms of control coverage and the global efficacy, and to simultaneously assess the consequences of interventions in the various parts of the village. The efficacy of intervention was evaluated using the set of parameters' estimates providing the best fit to the data. It was complemented by a sensitivity analysis of the corresponding results to the parameter's estimates. Each parameter 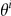 was then independently set to the boundary values of its confidence interval, i.e. 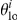 and 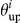, while keeping the others to their MLE.

We evaluated the efficacy of five types of control strategies applied individually, including indoor insecticide spraying, door and window insect screens, peri-domicile cleaning, triatomine lethal traps located in the peri-domestic habitat, and housing improvement to reduce house attractiveness to bugs. The effect of each strategy on bug survival, reproduction and/or dispersal was modelled as described below (see also supplementary methods — Text S1 — for the mathematical changes that were made to the model to include these effects).

**Indoor insecticide spraying** was modelled by reducing vector survival in each treated house as before [11]. The control-induced mortality was calculated with respect to the residual dose of insecticide that we adjusted daily, and to the lethality of the dose as expected from a typical sigmoid dose-response relationship. Assuming that the control-induced and natural mortalities act independently (i.e. to survive one of the two causes of death does not affect the probability to survive the second one), we combined them multiplicatively to define the overall survival probability.

We considered a spray rate of 50 mg.m^−2^ of pyrethroid insecticide at the beginning of the infestation season (since it was previously shown to be the optimal timing for spraying [11]), the half-life of the insecticide was set to 38 days, and the lethal doses 50% and 90% were fixed to 32.2 mg.m^−2^ and 182.4 mg.m^−2^ [11]. A sensitivity analysis to insecticide dose was performed predicting the effect of spraying at 100, 200 and 300 mg.m^−2^.

**Door and window screens** were considered as physical barriers impeding the arrival of a proportion of the non-domiciliated vectors into the domestic habitat, and were thus modelled by lowering immigration into the houses by a factor of bug exclusion *r* set at 85% and constant over time [11], [42]. Again, a sensitivity analysis was conduced by considering *r* equals to 70, 80 and 90%. Because the efficacy of screens is likely to depend on the behavioral response of dispersal bugs failing to enter houses because of screens, and because no information was available in the literature about such a response, we considered three alternative assumptions. Bugs that could not enter into houses were considered: (1) to stop dispersing and die, or (2) to stop dispersing for one day before starting again with no learning in their dispersal behavior (and thus possibly attempting to enter the same house), or (3) to go on dispersing while avoiding the house they could not enter.

**Peri-domicile cleaning** was assumed to eliminate all bug colonies established in this habitat for the rest of the current year. This reduced immigration from the cleaned sites, but did not have any effect on individuals that originated from other areas and may pass through the peri-domiciles where this control strategy was applied. In addition, we performed a sensitivity analysis by considering that cleaning removes only 60% and 80% of insects established in the peri-domestic habitat.

**Manipulation of houses' attractiveness** to bugs was achieved by decreasing *H* from its estimated value to 1, the value for which houses are no more attractive than the peri-domestic and sylvatic habitats. This represents the strongest possible effect and allows evaluating the maximal potential for this strategy; a sensitivity analysis for the intermediate values of *H* was then performed.

**Triatomine lethal traps** in the peri-domestic habitat were assumed to attract and kill triatomines into the cells where they are positioned according to an additional parameter *H_trap_* that measured the trap attraction. As for the study of the control of houses' attractiveness we first wanted to evaluate the maximal potential of this strategy. The density of traps was then fixed at 2 traps per household, and attraction was set to a constant level *H_trap_* = 12, almost twice the attraction of houses. Sensitivity analysis was then performed for different density of traps, in the range 5 traps per household to 1 trap for 10 households, and trap attraction, in the range 1 to 50.

## Results

### The model with no control

**Figure 2:**
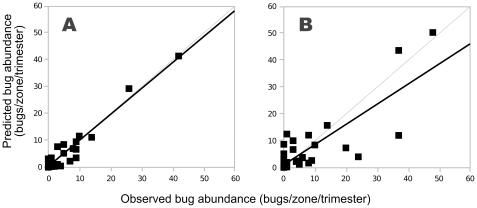
Correlation between observed and predicted bug abundance in the houses. (**A**) Descriptive value of the model: correlation between predicted values and abundance data during the first year of field collections that were used to estimate the model parameters. Predicted abundance = 0.97+0.36 x Observed abundance year 1 (McFadden's likelihood ratio index = .93). (**B**) Predictive value of the model: correlation between predicted values and the abundance data during the second year of field collections. Predicted abundance = 0.75+1.07 x Observed abundance year 2 (McFadden's likelihood ratio index = 0.67). The grey lines indicate a perfect relationship.

The model predictions fitted very well the yearly spatio-temporal dynamics of infestation observed in the village of Teya between mid-September 2006 and mid-September 2007. The correlation between observed and simulated spatio-temporal data indicated that the model reproduced well both the seasonal variations in triatomine densities, and the spatial spread of bugs from the border to the centre of the village (Figure 2A, McFadden's likelihood ratio index = 0.93). Importantly, the model parameterized with the data on this first year was able to predict the observed spatial and temporal dynamics of bug abundance in the following year (Figure 2B, McFadden's likelihood ratio index = 0.67). We note that while our model tends to predict well high abundances, predictions at lower vector abundances seem less precise. However, this is rather inconsequential since predicting fine variations in space and time at low abundances is of little relevance for our ultimate objective of evaluating control strategies.

The convergence of the presented results with a previous study, that used a stochastic model [24], also showed that the selected local and dispersal rules (see Definition of the function *f* including the *local* and *dispersal rules*) were reliable in their ability to both reproduce and anticipate the spatio-temporal dynamics of these non-domiciliated vectors. Likelihood profile confidence intervals gave further information on the estimated parameters of these rules (Table 1). Those confidence intervals were quite narrow around the MLE. The lower and upper boundaries were typically located at less than 30% of the MLE of each of the parameters, indicating that larger changes in one of the parameter estimates would no longer allow properly reproducing the data. The survival rates in the domestic and peri-domestic habitats were very close to 0.2 and 0.9, respectively; the numbers of insects immigrating from the colonies established in the sylvatic and peri-domestic habitats were in the range 150–260 insects for 15 days; there was nearly a 1∶1 ratio between immigration from the sylvatic and peri-domestic habitats; the attraction to the house was always at least 5 times higher than attraction to the peri-domestic area, and the optimal (and mean) distance of dispersal was between 50 and 60 meters (Table 1). All of those results were consistent with and supported our previous conclusions that insects found in houses came in roughly similar proportion from the sylvatic and peri-domestic habitats and that they disperse over rather small distances and with a strong attraction to the domestic habitat [24]. Overall, our spatial model with no control thus offered a good framework where spatially targeted control strategies could be evaluated.

**Table 1:** Maximum likelihood estimates (MLE) and 95% profile-likelihood confidence interval of the parameters of the model with no control.

Parameter^1^	MLE	95% CI
Adult survival in the domestic habitat *S_d_* (probability.time^−1^)	0.21	[0.17, 0.25]
Adult survival in the peri-domestic habitat *S_p_* (probability.time^−1^)	0.93	[0.91, 0.95]
Adults leaving peri-domestic colonies *K_p_* (number.time^−1^)	212	[180, 246]
Adults leaving sylvatic colonies *K_s_* (number.time^−1^)	203	[157, 259]
Rate of flight/walk initiation *d* (probability.time^−1^)	0.27	[0.19, 0.36]
Dispersal distance mode *D* (m per displacement)	53.2	[50.9, 55.5]
Standard deviation of the dispersal distance *σ* (m per displacement)	1.05	[1.0, 3.8]
House attractiveness *H* (no dimension)	6.6	[5.2, 8.8]

Estimates of *S_d_*, *S_p_*, *K_p_*, and *K_s_* are given for a 15 days period (to allow direct comparisons with previous estimates published in [24]), while *d* is given for a 1-day period (the time step of the model) as it is not linearly scaling with time.

### Efficacy of spatially targeted single control strategies

**Figure 3:**
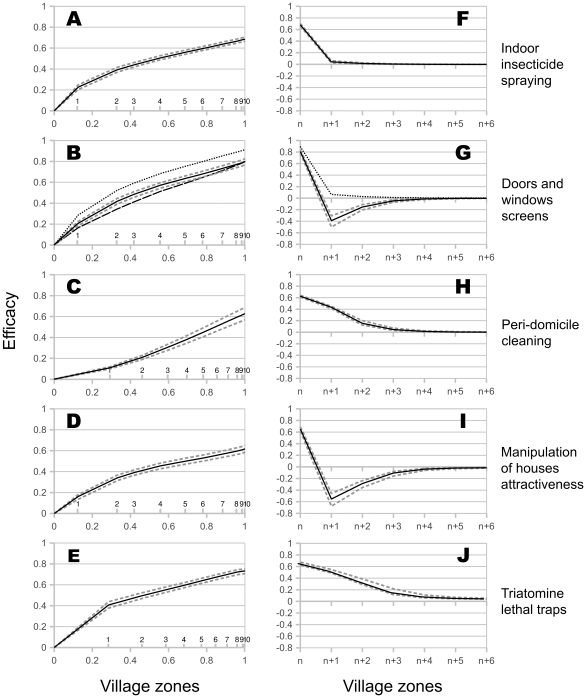
Global and local efficacy of the five control strategies. (**A–E**) Global efficacy. Relationships between the post-intervention reduction in bug abundance in the whole village, and the control effort, measured as the extent of village zones treated, expressed in proportion of the number of houses in the whole village (below the axis) and in number of village zones (above the axis). (**F–J**) Local efficacy. Reduction of bugs abundance in untreated village areas (>*n*) when the control intervention is limited to the *n* outer village zone(s). In A–J, grey dashed lines give the minimal and maximal efficacies obtained when demographic parameter values were varied within the 95% confidence interval of their MLE. In B and G, dashed, solid and dotted black lines correspond to the variation of efficacy according to the different assumptions made on bug behaviour after they fail to enter a given house because of screens. Dotted line: assumption 1, to stop dispersing and die. Solid line: assumption 2, to stop dispersing for one day before starting again with no learning in their dispersal behaviour (and thus possibly attempting to enter the house they just failed to infest). Dashed line: assumption 3, to go on dispersing while avoiding the house they could not enter. In G the dashed line is confounded with the solid one.

We investigated the efficacy of the five strategies considered independently by applying them to concentric rings defined from the border of the village and whose size was increased until a complete coverage of the village was reached. For each strategy, we calculated its efficacy, measured as the post-intervention reduction of bugs' abundance in the whole village, in function of the extent of village zones treated, i.e. the effort in terms of the control intervention (Figure 3A–E). We also calculated the effect of the interventions in each concentric village area, including those without control intervention (Figure 3F–J). Finally, we performed a sensitivity analysis to the parameter values by independently replacing the MLE with the upper and lower values of each profile likelihood based confidence interval (Table 1).

The first key point is that all the results obtained with each of the five strategies were only weakly sensitive to changes in demographic parameters values. Such changes indeed lead to no qualitative change in the form of the relationships (Figure 3). As expected, parameters with the strongest effect depend on the control strategy considered. Maximal changes were obtained when changing survivals (*S_p_*, *S_d_*) for insecticide spraying, immigration rates (*d*) for screens and outdoor traps, houses' attraction (*H*) for the control of houses' attractiveness and the number of individuals leaving colonies (*K_p_*, *K_s_*) for peri-domestic cleaning (results not shown). However, these effects were systematically lower than 5% on both treated (Figure 3A–E) and untreated areas (Figure 3F–J). The results obtained are thus very robust to variations of the parameters of the model with no control, and we will thus further describe only the results obtained with the MLEs.

**Indoor insecticide spraying** in the whole village allowed the reduction of total bug abundance over a year by about 70% for one year (Figure 3A). The relationship between the proportion of treated houses and global efficacy was a slightly convex diminishing return curve, so that half of the maximal decrease could be obtained by spraying only the first two external zones of the village (a third of the houses). We also evaluated the local efficacy of insecticide in untreated village zones at the forefront of the treated areas. Independently of the number of village areas sprayed, the use of indoor insecticide only reduces the vector abundance in the treated area; it has a negligible effect on neighboring untreated areas (Figure 3F). To increase the dose applied allowed the predicted levels of vector reduction to reach higher levels (doses of 100, 200 and 300 mg.m^−2^ lead to a 79%, 85% and 87% maximal control efficacy, respectively; data not shown), with no change in the main conclusion: Insecticide spraying in only the first two outer zones allowed for half of the maximal control efficacy.

**Door and window insect screens** applied to all the houses of the village decreased the total vector abundance by about 80% when bugs that could not enter into houses were assumed to go on dispersing (assumptions 2 and 3, the former including possible attempts at entering again the house they just failed to infest) (Figure 3B). As for insecticide spraying, there was a slightly convex diminishing return between the number of treated zones and efficacy. Accordingly, limiting the intervention to the first two zones at the periphery of the village (a third of the village houses) again led to half of the maximal reduction in abundance. Under the two assumptions not including the death of the insects failing to enter the houses [2]–[3], the analysis of insect screens' local efficacy indicated that while infestation was well controlled in houses with screens, the control had a detrimental effect on the immediate non-equipped neighbor: an increase of up to 40% in vector abundance was estimated in the most proximate untreated village zone (Figure 3G). This negative effect on neighboring areas disappeared for untreated areas more than 3 zones away from the treated one. On the other hand, when the vectors were assumed to die when failing to enter a house (assumption 1), the effects of screens were significantly different. In this case, vector abundance was reduced slightly further (up to 90%) when screens were used in all the houses of the village (Figure 3B upper dotted black line), and the control strategy then had no negative effect on untreated neighboring houses (Figure 3G upper dotted black line). To vary the efficacy of screens produced only small linear changes in the global efficacy. Under assumptions 2 and 3, a reduction factor *r* of 70%, 80% and, 90% lead to a 51%, 64% and 80% maximal control efficacy, while under assumption 1, a reduction factor *r* of 70%, 80% and 90% led to a 73%, 82% and 91% maximal control efficacy; data not shown. The above conclusions are consequently very robust to variations of *r*, which is thought to be in the range 80–90% in the field [42].

**Peri-domicile cleaning** reduced total bug abundance by up to 62% for one year when performed in the whole village (Figure 3C). The increase in efficacy with increasing coverage was a concave relationship with a slightly increasing return. Because of the lower efficacy of peri-domicile cleaning at the periphery of the village, intervention in at least the first 3 zones (60% of the village peri-domestic surface) was required to reach half of the maximal reduction in abundance. Interestingly, when peri-domicile cleaning was performed only in some parts of the village it had an important beneficial effect on untreated neighboring houses. The vector abundance in the two closest non-treated zones was reduced by 40% and 15% respectively (Figure 3H). Lowering the rate of colonies' destruction by peri-domicile cleaning, which was initially set to 100%, lowered the total efficacy in an almost perfectly linear way, but again had no effect on the above qualitative conclusions. Typically, assuming that only 80% or 60% of colonies are removed by cleaning peri-domiciles allowed for a maximal control efficacy of 50% (≈62%×80%) and 37% (≈62%×60%), and in both cases intervention in the first 3 zones was needed to get half of these outcomes.

**Figure 4:**
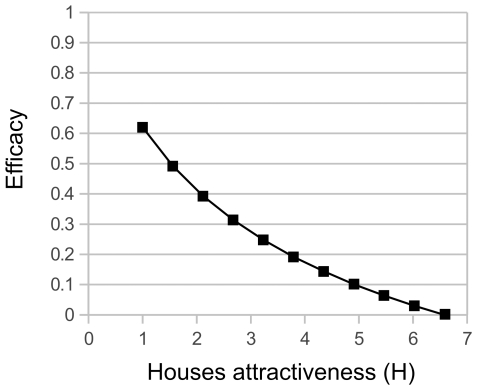
Efficacy of control by manipulation of houses attractiveness. Efficacy was measured as a percentage reduction in vector abundance in the whole village, when manipulating the attractiveness in all the houses of the village. Attractiveness was decreased from *H* = 6.6, its estimated value in absence of control (see Table 1), to *H* = 1 where houses were no longer attractive since they had the same *H* factor as peri-domestic areas.

**Manipulation of houses**'** attractiveness** was found 60% effective when applied to the whole village and when such attraction was completely eliminated, so that domestic habitat was no more attractive than the peri-domestic and sylvatic habitats (*H* = 1) (Figure 3D). Half of the maximal efficacy could be reached by an intervention targeted on the first two zones of the village representing a third of the village houses. However, such strategy had an important negative impact on the abundance of bugs in non-manipulated neighboring houses when applied to parts of the village (Figure 3I). Indeed, the lack of attraction of manipulated houses resulted in an increase of over 50% and 30% in bug abundance in the next two untreated village zones. Importantly, sensitivity analysis of intermediate values of reduction in house attractiveness indicated that efficacy of the intervention was rapidly lost as *H* was incompletely reduced: the maximal efficacy was of 40%, 17% and less than 5%, for *H* values of 2, 4, and 6, respectively (Figure 4).

**Insect lethal traps** were found potentially able to reduce global vector abundance by up to 72% when considering a high density (two traps per household) and a high attractiveness (*H_trap_* = 12, nearly twice the attractiveness of houses) and 100% of lethality (Figure 3E). Under these conditions, an important diminishing return was observed since to install traps in the first zone of the village (27% of the peri-domestic surface) allowed to attain half of the maximal efficacy. Furthermore, this strategy had substantial positive effects on the 4–5 neighboring areas without traps, where insects' abundance was decreased by 50%, 30%, 15% and 7%, respectively (Figure 3J).

**Figure 5:**
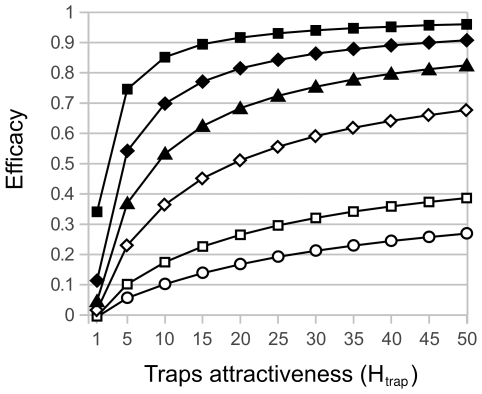
Efficacy of triatomine lethal traps according to their potential attractiveness and density in the village. Efficacy was measured as a percentage reduction in vector abundance in the whole village, when traps were installed all across the village. Attractiveness was increased from *H_trap_* = 1, where traps are not attractive since they had the same *H* factor as peri-domiciles, to *H_trap_* = 50. Symbols stand for different density of traps. Open circles: 1 trap for 10 houses, open squares: 1 trap for 5 houses, open diamonds: 1 trap for 2 houses, closed triangles: 1 trap per house, closed diamonds: 2 traps per house and closed squares: 5 traps per houses.

However, reducing the attraction factor of each trap had an important effect at the village scale as the global control went down from 72% to 55% when attraction of individual traps was reduced from *H_trap_* = 12 to *H_trap_* = 5, a value similar to the attractiveness of houses (Figure 5). On the contrary, to increase attraction to higher levels had almost no effect whatever the number of traps considered. To lower the number of traps also had a strong detrimental effect, and the reduction of bug abundance due to control was never found larger than 30% when the number of traps was dropped to 1 trap for 10 households (Figure 5). A nearly 100% control efficacy at the village scale was reached only when more than 2,500 traps were used in the village, which represent about 5 traps per household within the village.

#### Combined spatially targeted control strategies

**Figure 6:**
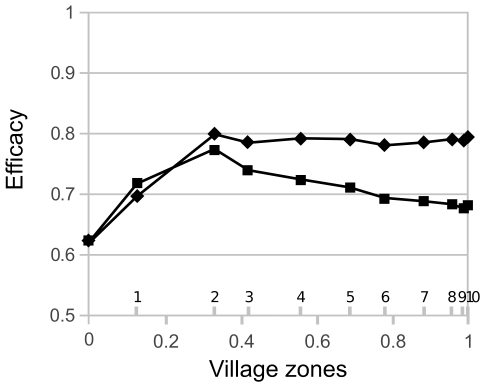
Efficacy of spatial combinations of vector control strategies. Insecticide spraying (squares) or insect screens installation according to assumption 2 (diamonds) in the inner part of the village are combined with peri-domestic cleaning in the remaining inner part. The x-axis gives the number of zones controlled with insecticide or screens (above the axis) and the fraction of the effort necessary to control the whole village with these strategies (below the axis). Efficacy was measured as a percentage of reduction in vector abundance in the whole village.

The above results suggest spatial combinations of strategies may improve control efficacy. We thus evaluated a combination of either insect screens (according to assumption 2) or insecticide spraying in the outer part of the village, to target vectors originating from both peri-domestic and sylvatic sources in this area, with peri-domicile cleaning in the centre, where peri-domestic colonies represent the major source of vectors infesting houses. The spatial coverage of each strategy was systematically varied to identify the optimum spatial combination. Combining either the use of insect screens or insecticide spraying in the first two outer zones of the village with peri-domestic cleaning in the centre, allowed an optimum reduction in vector abundance of up to 80% (Figure 6). Installing screens on larger parts of the village while reducing the coverage of peri-domicile cleaning did not provide any additional benefit and spraying on larger parts of the village controls less optimally (Figure 6). The maximum of efficacy observed for spraying in the two outer zones and peri-domestic cleaning in the inner zones is induced by the high proportion of insects from peri-domestic colonies in the inner areas; these insects are better controlled by removing the colonies than by the fast decaying insecticide, no more efficient at the end of the migration period.

#### Cost-effectiveness of individual and combined control strategies

Based on the potential cost of the different interventions [42], insecticide spraying in the entire village would quickly become expensive, as spraying needs to be performed every year [27], [42]. As for insect screens, although a complete coverage of the village could control vectors effectively over a longer period of time, the global cost remains high (Table 2). Importantly, spatial targeting of combinations of strategies can maintain (or even increase) vector control efficacy compared to single interventions, while significantly reducing cost. Indeed, either insecticide spraying or insect screens applied to houses in the two outer zones of a village, combined with peri-domicile cleaning in the centre, would provide optimum vector control at the lowest cost (Table 2).

**Table 2:** Cost-effectiveness of individual and combined vector control strategies.

Strategy	Efficacy in all village(% of reduction in vector abundance)	Cost for 1 year ($)	Cost for 5 years ($)
Insecticide spraying in all village^a^	70%	2,640	13,200
Insect screens in all village^b^	80%	21,600	21,600
Peri-domicile cleaning in all village^c^	60%	2,640	6,600
Insecticide spraying 2 zones in periphery+peri-domicile cleaning 8 zones in centre	80%	2,640	8,841
Insect screens 2 zones in periphery+peri-domicile cleaning 8 zones in centre	80%	9,078	9,078

a Insecticide spraying needs to be annual [9], [11]. Cost estimates include materials and labour, but not transportation to the villages.

b Efficacy of insect screens is given for assumption 2 and insect screens are assumed to last for 5 years.

c Peridomicile cleaning needs to be repeated every 2 years [42].

## Discussion

Although the elimination of transmission of Chagas disease was targeted by the WHO for the year 2010 [4], there are still large regions with active vectorial transmission mostly due to non-domiciliated triatomines [6]. These vectors do not constitute permanent colonies inside houses, so that domestic populations actually are typical ‘sinks’ sustained by peri-domestic and/or sylvatic ‘source’ populations [8]. The risk of transmission associated with these non-domiciliated vectors is thus now identified as a major challenge for the future of Chagas disease control [5], and a key objective is to evaluate the efficacy of classical or alternative control strategies to reduce their abundance. Since non-domiciliated insects infesting houses typically come from the sylvatic and peri-domestic habitat [11], to evaluate the potential of various strategies requires a good understanding of the village infestation dynamics in absence of control. In this perspective, spatial population dynamic models able to reproduce and predict the dispersion of individuals from these two non-domestic habitats are valuable tools.

Taking advantage of previous field and modelling works on well-studied populations of non-domiciliated triatomines in villages of the Yucatan Peninsula, Mexico, we performed the first attempt to evaluate the efficacy of putative control strategies applied spatially. We identified triatomines' dispersal characteristics through a selection model approach based on maximum likelihood estimates [37], [38]. The best deterministic model and the associated estimates of the dispersal characteristic identified here were found very similar to the ones identified in a similar approach, but based on stochastic models [24]. In addition, just as the previous more complex stochastic model, our deterministic model reproduced and predicted very well the spatio-temporal dynamic of the village infestation. The present study thus confirmed that the selection model approach is a well-adapted strategy to simultaneously obtain indirect estimates of triatomines dispersal, hard to quantify in the field [19], and robust GIS-based Spatially Explicit Models (GIS-SEM) able to reproduce and predict the dynamic of infestation in the absence of control. Such a model is required for the evaluation of the efficacy of putative control strategies; to this end we combined our selected model with a representation of different strategies to evaluate their potential.

We found that indoor insecticide spraying and insect screens applied to the entire village were able to reduce yearly vector abundance in the whole village by 70 and 80%. Interestingly, in both cases, half the maximal effect was obtained while interventions were limited to the first two outer zones of the village. This mostly reflected the higher abundance of insects typically found in houses in the periphery of the village, where the vectors dispersing from both the peri-domestic and sylvatic habitats contribute to domestic infestation [23], [24], [26].

Although global efficacy was roughly similar for these first two strategies, a possible difference between them could be on their effect on untreated neighboring households. Indeed, insect screens were shown to impose some additional infestation on nearby untreated houses when vectors were allowed to go on dispersing after failing to infest a protected house. However, this negative effect was not present when vectors were assumed to systematically die after their first attempt to infest a protected house. Interestingly, the latter scenario is qualitatively consistent with a field trial conducted in a village of the north of the Yucatan Peninsula, in which the use of impregnated curtains and windows screens in some houses seems to reduce bug abundance in nearby untreated houses [42]. This may be due to some knockout effect of the low dose insecticide used for impregnation, or to a poor energetic status and/or exhaustion of bugs that could prevent re-departure after a flight/walk to intent infest a first house.

Particularly in these conditions, and even if more empirical and modelling studies are needed to quantify vector dispersal at the individual scale, our results do support the idea of a spatially targeted use of insect screens to control the higher bug abundance at the periphery of the village as it maximizes the overall reduction in transmission risk at the level of the entire village. The most cost-efficient intervention would then be to treat the houses located in the first two outer zones (about 33% of the total houses of the village) to obtain around 50% bug abundance decrease in the entire village.

The weak effect of insecticide spraying on the neighboring houses shown in this study is also consistent with a field trial [42]. Treating the first two outer zones would allow obtaining about 40% decrease in total bug abundance in the entire village but with no efficiency on untreated areas. Those results suggest that the cost associated to the temporary effect of insecticide spraying on non-domiciliated vectors demonstrated at the house scale [9], [11], can only weakly be compensated for by spatially targeted strategies that would exploit the typical gradient of abundance due to the immigration of sylvatic bugs [26], [43].

Peri-domicile cleaning appears to be an interesting alternative strategy having the potential to substantially reduce vectors abundance inside the treated zones and to exert a positive influence on untreated areas. By eliminating all the colonies established in the backyards, a perfect cleaning of the peri-domiciles provided a 60% reduction of bug abundance in the village, although it provided a substantially lower efficacy at the periphery of the village, compared to the efficacy of residual insecticides and insect screens. This lower predicted efficacy at the village scale and in the outer zones is due to the absence of impact of this strategy on insects dispersing from the sylvatic habitat. It is also consistent with previous estimates indicating that infesting bugs come from both peri-domestic and sylvatic sites, and that both sources need to be controlled [24], [44].

Interestingly, the positive effect of this strategy on nearby households with no intervention confirmed results of a previous field trial where peri-domicile cleaning (elimination of unnecessary objects of the peri-domicile followed by insecticide spraying) also reduced infestation in neighboring houses without intervention [42]. Accordingly, peri-domicile cleaning could valuably be used to significantly reduce bug abundance, especially in the centre of the village where the majority of non-domiciliated vectors found in houses come from the peri-domestic habitat [24]. Because it targets specifically peri-domestic vectors, such a strategy could lead to a substantial level of control when combined with insect screens in the periphery of the village.

The manipulation of house attractiveness was explored here as a potential novel vector control intervention based on the rationale that triatomines were found to be directly attracted to the houses [24]. We found that such an intervention could reduce domestic infestation by up to 60% when applied to the entire village. However, when applied to only a fraction of the houses, we show that it would induce an increased infestation of neighboring untreated areas as bugs no longer attracted to manipulated houses tend to disperse to nearby domiciles. Control intervention based on this strategy should thus preferentially be implemented in all the houses of the village, and feasibility would then rely on the kind of modifications to be done in the domestic habitat to limit attraction.

The actual determinants for house attractiveness to bugs are still unknown, but if light is proven to be a key factor [28], [45], [46], the use of devices limiting the diffusion of the light may be considered. Nevertheless, it is important to emphasize that the effect of the intervention is rapidly lost if the reduction in the attractiveness is only partial. This strategy would thus be of little interest if a nearly complete reduction in house attractiveness to bugs could not be achieved. Thorough research on the mechanism and factors of triatomine dispersal toward houses would then be needed to allow the implementation of such a strategy.

Triatomine lethal traps were also tested in an attempt to keep bugs away from the houses. Such traps were estimated effective if they were highly attractive and lethal, and used at very high densities; in these conditions they would also have a marked beneficial effect on neighboring houses without traps. The attractiveness of potential traps such as yeast-baited traps is difficult to estimate, but available studies suggest an attractiveness *H* in the range of 2–3, i.e. rather less that the attractiveness of houses evaluated at 6–7 [47]–[49]. In such conditions, the use of 5 traps per household, which would represent about 2,500 traps in the whole village, only allows for about 30% reduction of triatomines abundance in the village.

In addition, traps were assumed to be of constant efficacy in our model, which seems to be highly unlikely in practice, as it would raise the issue of the periodic maintenance/renewal of the traps depending on their half-life. It thus seems that the performance of potential outdoor traps would need to be dramatically improved to become a viable strategy for non-domiciliated triatomine control.

Overall, this study has shown that control strategies applied at the periphery of a village can contribute to reduce infestation in untreated, more central houses, but only in limited proportions. Typically, insecticide or insect screens used in the first two outer zones of the village, which represents 33% of the households, would only reduce vector abundance in the whole village by 40–50%. In these conditions, spatial targeting of strategies based on either insecticide spraying or insect screens applied to houses in the two outer zones of a village, combined with peri-domicile cleaning in the centre, would provide optimum vector control at the lowest cost (Table 2). Essentially, such mixed strategy would remove peri-domestic colonies where they are the major source of vectors, and impede the insects to enter houses where they also come from non-manageable sylvatic colonies.

At first, the costs of combining insecticide spraying or insect screens with peri-domicile cleaning seem roughly equivalent. However, the seasonal pattern of house infestation requires, for insecticide spraying, the dispatch of a large number of spraying teams to cover an entire region within 2 months [11], generating additional costs of transportation and logistics [27].

Thus, a combination of insect screens in the periphery and peri-domicile cleaning in the centre would be the most cost-effective and sustainable strategy to be implemented in the Yucatan Peninsula. The design of such spatially mixed strategies of control offers a promising avenue to reduce the economic cost associated to the repeated intervention intrinsically associated with the permanent re-infestation of houses by non-domiciliated vectors [9], [11].

## Supporting Information

Alternative Language Author Summary S1 Alternative Language Author Summary in Spanish translated by Eric Dumonteil.
(0.03 MB DOC)

Text S1 This appendix includes the equations modeling the dispersal dynamics and control of vectors.
(0.06 MB DOC)
