# Supplementary material for: Evaluation of Spatially Targeted Strategies to Control Non-Domiciliated Triatoma dimidiata Vector of Chagas Disease
Source: PLoS Negl Trop Dis. 2011 May 17;5(5):e1045. doi: 10.1371/journal.pntd.0001045 (PMC3096612; doi:10.1371/journal.pntd.0001045)
Supplement: Alternative Language Author Summary S1 — Alternative Language Author Summary in Spanish translated by Eric Dumonteil. (0.03 MB DOC) [file pntd.0001045.s001.doc]

# Author Summary in Spanish

La enfermedad de Chagas es una de las enfermedades parasitarias mas desatendida en America Latina. desde los años 1980, varias iniciativas nacionales e internacionales han contribuido a la eliminación de los vectores presentes en las viviendas. El reto actual es el de controlar los vectores que no están adaptados a las viviendas, pero que son capaces de transmitir el parasito durante incursiones cortas en ellas. En este estudio, evaluamos el potencial de diferentes estrategias de control vectorial aplicadas según patrones espaciales definidos, gracias a un modelo matemático que reproduce la dinámica de dispersión de estos vectores no-domiciliados en una localidad de la península de Yucatán, México. Demostramos que ninguna estrategia aplicada en la periferia de la localidad, donde los insectos son mas abundantes, brinda una protección satisfactoria a toda la localidad. Sin embargo, una combinación de mosquiteros en las casas de la periferia (para combatir insectos que se dispersan tanto de la selva como de los patios) y de la limpieza de los patios en el centro de la localidad (donde los insectos selváticos están ausentes), podría brindar un control efectivo. Este tipo de estrategia mixta parece muy prometedora para reducir el costo asociado a las intervenciones repetidas que requiere el control de los vectores no-domiciliados que intentan constantemente incursionar en las viviendas.
