# Supplementary material for: Evaluation of Spatially Targeted Strategies to Control Non-Domiciliated Triatoma dimidiata Vector of Chagas Disease
Source: PLoS Negl Trop Dis. 2011 May 17;5(5):e1045. doi: 10.1371/journal.pntd.0001045 (PMC3096612; doi:10.1371/journal.pntd.0001045)
Supplement: Text S1 — This appendix includes the equations modeling the dispersal dynamics and control of vectors. (0.06 MB DOC) [file pntd.0001045.s002.doc]

# Supplementary Methods

**Modelling of indoor insecticide spraying.** Indoor insecticide spraying was modelled by reducing vector survival in each treated house according to the dose of insecticide present in these houses, and the expected lethality of the dose. In any treated cell c, the quantity $q_{\text{ins}}(c,t)$ of residual insecticide was adjusted daily to account for an exponential decay of the active ingredient as follows:

(A1)

where *t_1/2_* and *Q*(*c*) stand for the half-life of the insecticide, and the dose applied in cell *c* at *t*=0, respectively. The effect of the dose $q_{\text{ins}}(c,t)$ on survival probability in cell *c* was determined according to a classical sigmoid dose-response relationship:

(A2)

where represent the hill slope of the sigmoid and *LD*_50_ and *LD*_90_ stand for the doses that kills 50% and 90% of individuals in 24h, respectively. Assuming that the natural and control-induced mortalities act independently, they were combined multiplicatively to define the overall survival probability. When simulating control strategies including indoor insecticide spraying, equation 4 was thus replaced by

(A3)

in any cell *c* of *A_dti_*, the subset of the domestic cells that were treated.

**Modelling doors and windows insect screens**. Insect screens were modeled by lowering immigration by a factor of bug exclusion *r*. When control strategies involving insect screens were simulated, equation 5a was thus replaced by

(A4)

in any cell *c* of *A_dts_*, the subset of the domestic cells that were protected. When insects failing to enter a house were assumed to die (first assumption in the paragraph ‘Evaluation of spatially targeted strategies – Doors and windows screens’ of the main text), this change was the only change made in the model. When insects failing to enter a house were assumed not to die but stopping their dispersion for this time step (second assumption in the paragraph ‘Evaluation of spatially targeted strategies – Doors and windows screens’ of the main text), they were placed randomly in one of the peri-domestic cells surroundings the domestic cells they just failed to enter. The dispersal rule given by equation 5a was then changed also for any peri-domestic cell *c* surrounding cells of *A_dts_* to include the influx of bugs that could not enter into the protected houses because of screens. Equation 5a was then replaced in this additional set of cells by:

(A5)

where *v*(*c*) denotes the Moore neighborhood of range 1 for cell *c*.

Under assumption 3 (learning in the bug dispersal behavior) equation A(4) was first applied but the insects failing to enter the houses were at the same time step redistributed in the neighborhood *v*(*c*) of the house they failed to enter following the same rules than insects initially in the cell *c* and avoiding it.

**Modelling peri-domicile cleaning**. Peri-domicile cleaning was assumed to allow removing colonies established in the peri-domestic habitat. When control strategies including peri-domicile cleaning in a subset *A_pt_* of the peri-domestic habitat were simulated, *K_p_* in equation 5a was replaced by :

(A5)

for any cell *c*’ ∈ *A_pt_* of the domestic or peri-domestic habitats with *e* the efficiency of peri-domestic cleaning (rate of elimination of insects in peri-domestic colonies).

**Modelling manipulation of the attractiveness of houses**. The parameter *H* quantifying house attraction contributes to determine the values of the probabilities $p_{c'c}$ that appear in equation 5a. The equation itself was thus not modified, but probabilities $p_{c'c}$ were recalculated according to the value H that was used.

**Modelling attraction by traps in the peri-domestic area**. Traps were modelled by considering that they increase the attraction to the peri-domestic cells were they are located, and that they capture and kill all individuals that disperse to the corresponding cells. A parameter of attraction H_trap_ was defined for the cells of the peri-domestic habitat where such control is applied. Again, the equation 5a was thus not modified, but probabilities $p_{c'c}$ were recalculated according to the value H_trap_ that was used.
